# MWCNT-coated textiles for heating and temperature sensing under varying environmental conditions

**DOI:** 10.1039/d5ra07103h

**Published:** 2025-12-08

**Authors:** Babak Abdi, Esubalew Kasaw Gebeyehu, Ali R. Tehrani-Bagha

**Affiliations:** a School of Chemical Engineering, Aalto University Espoo 02150 Finland ali.tehrani@aalto.fi

## Abstract

In this study, a conductive cellulosic fabric was developed using a simple knife-edge coating method with multiwall carbon nanotubes (MWCNT) as conductive fillers and two different binders. The coated fabric was evaluated for its potential as an electrical heating textile and a temperature sensor. The investigation focused on the impact of environmental factors, including relative humidity and ambient temperature on the electrical heating performance of the coated textile samples. Increasing bio-based ink layers improved conductivity from 292 ± 38 S m^−1^ for three-layer coating to 623 ± 120 S m^−1^ for four-layer coating. IR thermal imaging revealed rapid Joule heating, with surface temperatures reaching 53 °C at 10 V. Relative humidity (RH) significantly influenced heating behavior, with peak surface temperatures observed at 70% RH, which was roughly 13 °C higher than that at 10% RH. While higher RH improved maximum surface temperatures, it also reduced initial heating rates. Conversely, lower ambient temperatures led to greater heat dissipation, reducing overall surface temperatures. We evaluated the electrical heating properties of the coated fabrics both without insulation and with different insulating layers, including cotton, viscose, and wool fabrics. This study identified optimal insulating layers for wearable heating pads. Wool provided superior heat retention, while the smooth and pliable cotton fabric proved ideal for skin contact, ensuring efficient heat transfer and breathability. The coated fabric also functioned as an accurate temperature sensor, exhibiting a strong linear relationship between temperature and measured resistance under ambient conditions. The Temperature Coefficient of Resistance (TCR) values confirmed its reliability and repeatability during cyclic heating and cooling.

## Abstract 2

In this study, a conductive cellulosic fabric was developed using a simple knife-edge coating method with multiwall carbon nanotubes (MWCNT) as conductive fillers and two different binders.

## Graphical abstract

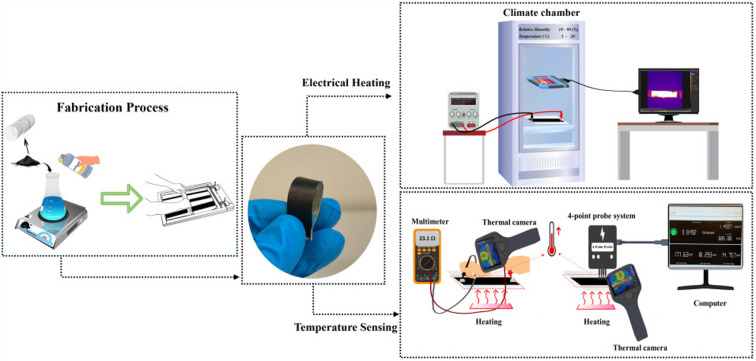

## 1. Introduction

Textiles play a crucial role in regulating body temperature across various environmental conditions.^1^ When the ambient temperature drops below body temperature, the body loses heat to the environment. Although metabolic processes work to maintain a core temperature of ∼37 °C, colder conditions increase heat loss, making it harder to retain warmth.^2^ Without adequate thermal protection, extreme cold can lower core body temperature, increasing the risk of hypothermia and reducing human performance and efficiency.^3^ Two main strategies to prevent body heat loss are using insulating textiles and applying external heating to the clothing microclimate. Textile-based heating materials are durable, flexible, and lightweight, making them ideal for cold-weather garments, bedding, and medical or therapeutic uses.^4^

The fundamental principle of heat generation through electrical resistance is defined in the joule heating concept. All electrically conductive materials possess resistance, which opposes the flow of electricity and, as a result, generates heat. Various electroactive materials such as carbon nanotubes (CNT),^5,6^ graphene,^7–9^ MXene,^10,11^ metallic nanomaterials,^12,13^ and conductive polymers^14,15^ can be embedded into textiles to form electrodes. Metal-based materials like gold, silver, and copper offer high heat generation at low voltages and are widely used in industry. However, their high cost, brittleness, and poor durability under washing and bending limit their suitability for flexible and long-lasting textile applications.^16,17^ In addition, the low mechanical strength of the conductive polymer fibers makes them vulnerable to physical damage from external forces or contact.^18^ On the other hand, carbon-based materials offer excellent conductivity, lower cost, biocompatibility and eco-friendly properties.^19–22^ This highlights the importance of expanding research on carbon-based conductors for fabricating electrical heating textiles.

Smart textiles research enables the integration of electronics into unconventional materials, driving innovation and expanding scientific frontiers. A key advantage is the ability to rapidly and efficiently functionalize large surface areas through established textile and electronics manufacturing processes.^23^ Recent advances in electronic textiles have enabled the integration of flexible, wearable sensors into fabrics, supporting continuous health monitoring. This progress has been key to developing wearable health monitoring systems that collect and transmit vital health data over extended periods. Body temperature is a key indicator of physical condition, influencing comfort, heat or cold stress, and overall performance.^24^ Temperature sensors have essential applications in everyday life, particularly in biomedicine, the food industry, and aerospace.^25,26^ Temperature sensors detect physical changes from temperature fluctuations and convert them into electrical signals. They come in various types based on their materials and operating principles, including resistance temperature detectors, infrared sensors, and thermocouples.^27^ This has driven extensive research into advanced sensing materials. Effective temperature sensors have traditionally relied on metal and metal oxide semiconductors.^28–30^ However, these sensors often face limitations such as rigidity, high density, and fragility.^27^

The rise of artificial skin^31,32^ and wearable electronics^33,34^ has intensified efforts to miniaturize sensors, enabling the development of soft and flexible materials. Most body temperature measurements in research use commercial thermistors and temperature ICs, typically attached externally to garments.^35^ Only a few studies have explored the development of temperature-sensing fabrics,^36^ and these were initial studies relying on complex manual fabrication processes. Therefore, there is still a significant research gap remains in fabricating flexible coated textiles for temperature sensing applications.

In our previous research studies, we showed the potential of using carbon-based conductors (MWCNT and graphene nanopowder) in bio-based coating formulations.^37^ The use of bio-based binders offers notable environmental benefits. Bio-based systems are derived from renewable resources and can contribute to reduced carbon footprint and improved biodegradability, aligning with green chemistry principles and sustainable material development. Our results indicated that CNT-based coatings demonstrated significantly superior electrical conductivity and Joule heating performance compared to graphene-based fillers in the same formulation, making them highly effective for the intended application.^37^ However, their potential health and environmental impacts remain a concern.^38^ To mitigate potential risks, the CNTs in this study were fully embedded within polymeric binder matrices, which substantially reduced the likelihood of particle release during handling or end use. We conducted detailed experiments on the comparison of the durability and electrical heating properties of the coated inks with a synthetic binder and a bio-based binder on a cellulosic fabric.^39^

Air humidity affects the moisture content in clothing, influencing heat and moisture transfer between the body, clothing, and environment, and thereby impacting comfort. For optimal thermo-physiological comfort in cold conditions, textiles should efficiently manage moisture transfer. This involves wicking away perspiration from the skin, facilitating its evaporation, and preventing the accumulation of dampness, which can lead to discomfort and heat loss. Proper moisture management ensures that the wearer remains dry and warm, enhancing both thermal insulation and overall comfort.^40–42^ Real-world environmental conditions often deviate from ideal scenarios, leading to potential discomfort for the wearer. Therefore, investigating the impact of factors such as relative humidity, ambient temperature, and the addition of insulating layers on the electrical heating properties of textiles is essential for optimizing their design and enhancing wearer comfort. This study aims to investigate the impact of external environmental factors, including relative humidity and ambient temperature, on the thermal performance of electrical heating textiles. Moreover, the insulation effect for heat preservation is investigated by using different types of fabrics as insulators on top of the coated patterns. This research enhances the understanding of performance and contributes to the improved design of electrical heating textiles.

## 2. Experimental

### 2.1 Materials

A knitted fabric comprising 70% rayon and 30% organic cotton, with a weight of 175 g m^−2^ and a yarn count of 60 Tex, was sourced from Coveross in Finland and was used as the coating substrate. A standard wool fabric no. 2664 with 100% wool Flannel and an aerial density of 268 g m^−2^ provided by TESTFABRICS, Inc., U. S. A. The fabric features a typical twill weave structure and a fully napped surface finish. The fabric had a plain weave structure with a thread density of 15 ends per cm and 15 picks per cm. A woven viscose fabric with an aerial density of 98 g m^−2^ from Linz Textile Company (Austria) was sourced. It was constructed using ring-spun yarns of 50/1 Nm in both the warp and weft directions, with a fabric width of 142 cm and an areal density of 98 g m^−2^. The thread density was 24 ends per cm in the warp and 21 picks per cm in the weft. These fabrics were used to investigate the insulation layer effect. Multi-walled carbon nanotubes (MWCNTs) featuring diameters of 50–85 nm, lengths of 10–15 µm, and a carbon purity above 94% were obtained from Graphene Laboratories Inc. The MWCNTs were used as received, with no chemical modification. The SEM analysis confirmed the tubular and worm-like particle agglomeration of MWCNT.^37^ OC-BioBinder™ Lily 1450 (OC), a water-based liquid with approximately 27% solid content, was provided by OrganoClick® AB in Sweden. The composition of the OC BioBinderTM Lily 1450 has been presented as l-(+)-lactic acid, (2*S*)-2-hydroxypropanoic acid with 1,2 benzisothiazol and 5-chloro-2-methyl-3(2*H*)-isothiazolone with 2-methyl-3(2*H*)-isothiazolone as preservatives.^43^ A polyurethane (PU) binder (A-5001) with an aroma-free, hydrocarbon-rich composition, specifically suited for textile printing, was sourced from Wennström in Finland. The chemical structure of the binders was characterized using Fourier-transform infrared spectroscopy, and the spectra have been provided in the SI (Fig. S1).

### 2.2 Fabrication process

#### 2.2.1. Coating ink formulation

In this study, the coating inks used in the fabrication process are consistent with those applied in our previous work.^39^ This ensures continuity and comparability in the performance and characteristics of the materials. Specifically, the inks provided suitable printing properties as well as high conductivity, flexibility and durability which are essential for the desired functionality in this application. For this process, an optimized MWCNT concentration of 16.7 wt% in two different binders was selected. To prepare the solution, MWCNT was first dispersed in 10 ml of water, stirred for 10 minutes, and then sonicated for 25 minutes at room temperature. Following this, the chosen binder (OC or A-5001) was gradually added to the mixture, and the binder concentration was calculated to be 8.3 wt% in both binder setups. Final inks were stirred continuously for 1 hour. After four successive coating layers, the total fabric weight gain ranged from 50.5 ± 2% for three different samples. Each layer contributed incrementally to the overall mass gain, with relatively uniform deposition ensured through controlled knife-coating parameters.

#### 2.2.2. Fabrication process

The coating procedure in this study closely follows the fabrication process used for the hybrid sample in our previous work, in which the coating was optimized.^39^ Here, the prepared inks were applied to a fabric in a rectangular pattern using a knife-edge coating technique. For this, an open frame was designed to form a 1 × 11 cm^2^ rectangular pattern, with the surrounding fabric covered to prevent ink spread. Four coating layers were applied in total:

(1) The first layer used ink containing the A-5001 binder, applied evenly with a squeegee through the open frame, then dried and cured at 150 °C for 3 minutes.

(2) Next, the bio-based ink was coated using the same technique, followed by drying at 120 °C for 3 minutes.

(3) An additional bio-based ink layer was applied, slightly adapting the previous procedure, with identical drying conditions.

(4) Finally, a second layer of A-5001 based ink was applied and cured at 150 °C for 3 minutes.

After the coating process, the samples underwent hot-pressing at 165 °C under 150 kPa for 10 minutes to ensure durability and consistency in the coating layers.

### 2.3 Characterization

#### 2.3.1 Thickness

The thickness of the coated samples was measured using a Lorentzen & Wettre SE 250 D thickness tester (Finland), with a micrometer resolution and an accuracy of ±1 µm.

#### 2.3.2 Bending test

The bending resistance and bending stiffness of the samples were evaluated using the Lorentzen & Wettre SE 160 Bending Tester (Finland). A bending length of 25 mm was used. Bending resistance was measured at a bending angle of 30°, while bending stiffness was determined specifically at 7.5°. Each sample was tested 10 times, and the average value was reported.

#### 2.3.3 Optical microscopy

The surface microstructure analysis of the layering fabrics was investigated using an Olympus BX53M optical microscope. The samples were monitored using 1.25×, and 5× objective lenses with both transmitted and reflected light illumination.

#### 2.3.4 Pore area measurement

Pore area measurements were conducted using optical microscopy combined with digital image analysis. First, high-resolution images of the fabric surface were captured using an optical microscope in reflected light illumination mode, and then the pore areas were calculated using ImageJ software. For this, the images were converted to 8-bit grayscale, and then, a consistent thresholding method was applied to isolate the pores. Finally, the software's particle analysis tool was then used to measure the pore areas in multiple fields of view, and the average pore area was calculated.

#### 2.3.5 Scanning electron microscopy (SEM)

The surface morphology and microstructure of the coated textile samples were analyzed using Scanning Electron Microscopy (SEM). Samples were cut into small sections and mounted on aluminum stubs with double-sided conductive carbon tape to ensure proper adhesion and conductivity. A thin gold/palladium (80/20) coating was applied to prevent charging and enhance image quality. SEM imaging was carried out using a Zeiss Sigma VP emission SEM (Germany) equipped with an InBeam detector, operated in high-vacuum mode at an accelerating voltage of 20 kV. Images were captured at various magnifications to assess surface morphology, coating uniformity, and particle distribution.

#### 2.3.6 Electrical conductivity

The resistance of each textile sample was measured using a BK391A digital multimeter along the entire 11 cm length. To ensure accurate readings, aluminum tape was applied to the sample ends, secured with silver paste for solid contact with the coated surface. Additionally, spot-by-spot electrical conductivity and resistance were tested with a four-point probe system (Ossila, UK). During the measurement, a current was applied between the outer probes, and the resulting voltage drop across the inner probes was monitored to determine the sheet resistance. The probes spacing were 1.27 mm apart. The system is capable of measuring between 100 mΩ sq^−1^ and 10 MΩ sq^−1^. In this method, 10 locations on each sample were measured, and the mean values were reported with standard deviation.^44–46^

#### 2.3.7 Electrical heating measurement

Ambient temperature and humidity were controlled using a RUMED Control 2000 climate chamber, set to vary ambient temperatures from 5 °C to 20 °C in 5 °C increments, and humidity from 10% to 90% in 20% steps. The Joule heating effect of the coated samples was tested under both constant ambient temperature with varying humidity and constant humidity with varying ambient temperature. The air velocity was controlled by the environmental chamber to maintain the set temperature and RH values. We did not set or control the air velocity during the measurements. To ensure proper connectivity, aluminum foil tape was applied to each sample's ends, secured with silver paste. Samples were conditioned in the chamber for one hour at each setting before measurements. While this time frame was sufficient for comparative purposes, it may not fully account for thermal and moisture equilibrium. Future studies will include extended conditioning periods to ensure more accurate evaluation under environmental influences.

For testing, a DC power supply (HY3005D) was connected to the samples, and surface temperature changes were recorded using a FLIR E60 infrared thermal camera, which has a temperature range of −20 °C to 650 °C, an accuracy of ±2 °C, and a thermal sensitivity below 0.05 °C. The camera was positioned at a fixed distance to capture the entire sample length, with a 25° angle relative to the horizontal plane to minimize IR reflection from the lens.

#### 2.3.8 Washing durability

The washing fastness of the coated samples was tested using a Testex TF418E instrument (China), which includes a temperature-controlled water bath and twelve rotating containers. Each container holds a stainless steel cylinder (75 ± 5 mm in diameter, 125 ± 10 mm in height, 550 ± 50 ml capacity) that rotates at 40 ± 2 rpm, with water temperatures adjustable from 0 to 100 °C.

The washing process adhered to the ISO 105-C10:2006 standard. This standard specifies a soap solution of 5 grams per liter, a liquor-to-sample mass ratio of 50 : 1, a water bath temperature of 40 °C, and a washing time of 30 minutes. Each sample underwent five wash cycles, with resistance and weight changes measured after each cycle.

#### 2.3.9 Thermal behavior of insulation layers by fabric touch tester

The tactile and thermal properties of the layer fabrics used in the insulation experiment were tested by SDL Atlas Fabric Touch Tester (FTT, M293). All samples were conditioned for 24 h at 20 ± 2 °C and 65 ± 5 °C relative humidity before the test. Samples were prepared in L shape, and fabrics were tested on both sides and in warp (a) and weft (e) direction according to the recommendation of the manufacturer.^47,48^ For each fabric side, three specimens were measured and the averaged results were presented. The indices that affect the transit and steady state heat conduction were used in the results to interpret warm feel and heat retention from the MWCNT-coated sample by the insulating layers and to analogize with the IR data.

#### 2.3.10 Moisture regain

The moisture regain of the insulating fabrics was evaluated at two different relative humidity levels, 50% and 90%. Wool, cotton, and viscose fabrics were placed inside a controlled humidity chamber set to the respective humidity levels for a specific time period, and their weights were recorded afterwards. Subsequently, the samples were dried in an oven at 110 °C for 30 minutes. After drying, the final weight was measured immediately, and the moisture regain for each fabric type was calculated using eqn (1). Although standard methods define oven-dry weight as the constant mass obtained by drying at 105 ± 3 °C until weight stability (within 0.05% over successive 20-minute intervals).^49^ This simplified method does not claim to reflect precise absolute moisture content but enables relative comparisons between sample types.1
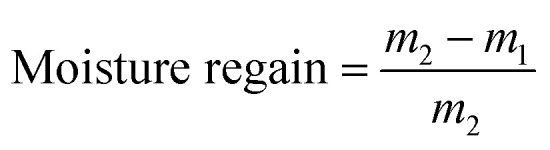
where *m*_1_ is the weight of the sample after 1 hour of constant relative humidity exposure, and *m*_2_ is the weight of the sample after drying.

#### 2.3.11 Temperature sensing

The temperature sensing was evaluated by analyzing the electrical responsivity to temperature changes through two separate procedures to show the spot responsivity and the whole sample responsivity. In the first procedure, the sample was heated under the 4-probe conductometer, and the surface temperature was monitored using the thermal camera. In the second procedure, the whole length of the sample was connected to the digital multimeter. Then, the whole area of the sample was heated, and the surface temperature was monitored by a thermal camera, and the change in resistance was recorded by the change in temperature.

## 3. Results and discussions

### 3.1 Fabrication and structural characterization

**Fig. 1:**
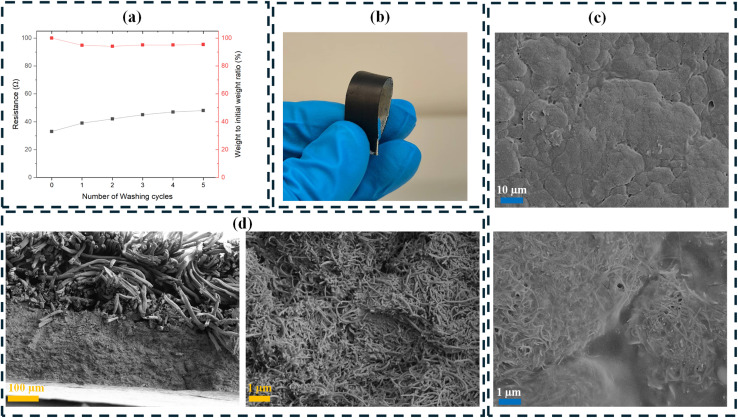
(a) Resistance and weight change after 5 washing cycles for the 4L hybrid sample, (b) surface image and demonstration of flexibility of the 4L hybrid sample, SEM image of the (c) surface and (d) cross-section of the 4L hybrid sample.

This study aimed to explore the impact of ambient temperature and relative humidity as key environmental factors, as well as the influence of insulation with different fabric layers on the electrical heating performance of coated fabrics. Additionally, it assessed the potential of these fabrics for temperature-sensing applications. Building on our previous work,^39^ where a hybrid approach demonstrated strong durability in electrical heating applications under washing, rubbing, and bending stresses, we adapted the process to fabricate a four-layer coated fabric in a rectangular shape (11 cm long and 1 cm wide). In this design, the first and fourth layers used ink with the A-5001 binder, while two central layers used bio-based ink. While, this approach still involves several of the challenges commonly associated with multi-step procedures, our objective was to increase the proportion of bio-based binder in the coating formulation with the long-term goal of developing fully bio-based electrical heating textiles. Moreover, this configuration was chosen to examine whether adjusting the middle layers could enhance electrical conductivity while maintaining durability. It can be seen that the addition of another coating layer of the printing layer using the ink containing the bio-binder has increased the mean electrical conductivity to 623 ± 120 S m^−1^ in comparison to the sample with three coating layers and a mean electrical conductivity of 292 ± 38 S m^−1^ (Table 1). The virgin fabric bending stiffness at a bending angle of 0–7.5° and bending resistance over a bending angle of 0–30° were measured as 0.031 ± 0.003 mN m and 2.6 ± 0.1, indicating its inherent softness and flexibility. After coating, the stiffness increased, reflecting the impact of the deposited conductive layers. Adding another coating layer increased bending resistance and stiffness, reducing flexibility. Specifically, bending resistance rose by 54%, while bending stiffness increased by 24%. The comparison of washing durability after five cycles in Fig. 1a revealed that adding an additional middle layer did not affect the durability of the hybrid-coated samples, which followed similar trends to the three-layer hybrid samples. Fig. 1b shows the surface image of the bent sample held between two fingers. Despite the addition of an extra coating layer increasing bending stiffness and resistance, the sample maintained high flexibility. Overall, hybrid-4L exhibited good conductivity, flexibility, and wash-fastness properties. Fig. 1c and d shows the SEM image of the surface and the cross-section of the coated 4L hybrid sample. The hybrid configuration enables the bio-binder to be sandwiched between two robust A-5001 layers. As observed in the SEM micrograph in Fig. 1d, the four layers are well-integrated and exhibit no clear separation between them, and these layers are practically indistinguishable from one another. This layered architecture promotes overall coating integrity and mechanical stability. As the A5001 layers provide anchoring to the fabric surface, while the bio-binder in the middle contributes to the environmental friendliness and electrical properties improvement without significantly compromising performance. The SEM image supports the hybrid hypothesis that this hybrid structure balances durability and sustainability in the coating system.^39^

**Table 1:** Electrical conductivity and characteristic properties comparison between 4L and 3L coated hybrid samples

Sample	Number of coating layers	Mean sheet resistance (Ohm per square)	Mean conductivity (S m^−1^)	Resistance (Ω)	Bending resistance (mN) (0–30°)	Bending stiffness (mN m) (0–7.5°)
Hybrid-4L	4	4.2 ± 0.9	623 ± 120	27.5 ± 5.5	20.6 ± 1.6	0.31 ± 0.04
Hybrid-3L	3	9.2 ± 1.1	292 ± 38	62	13.4 ± 0.6	0.25 ± 0.02

### 3.2 Joule heating

**Fig. 2:**
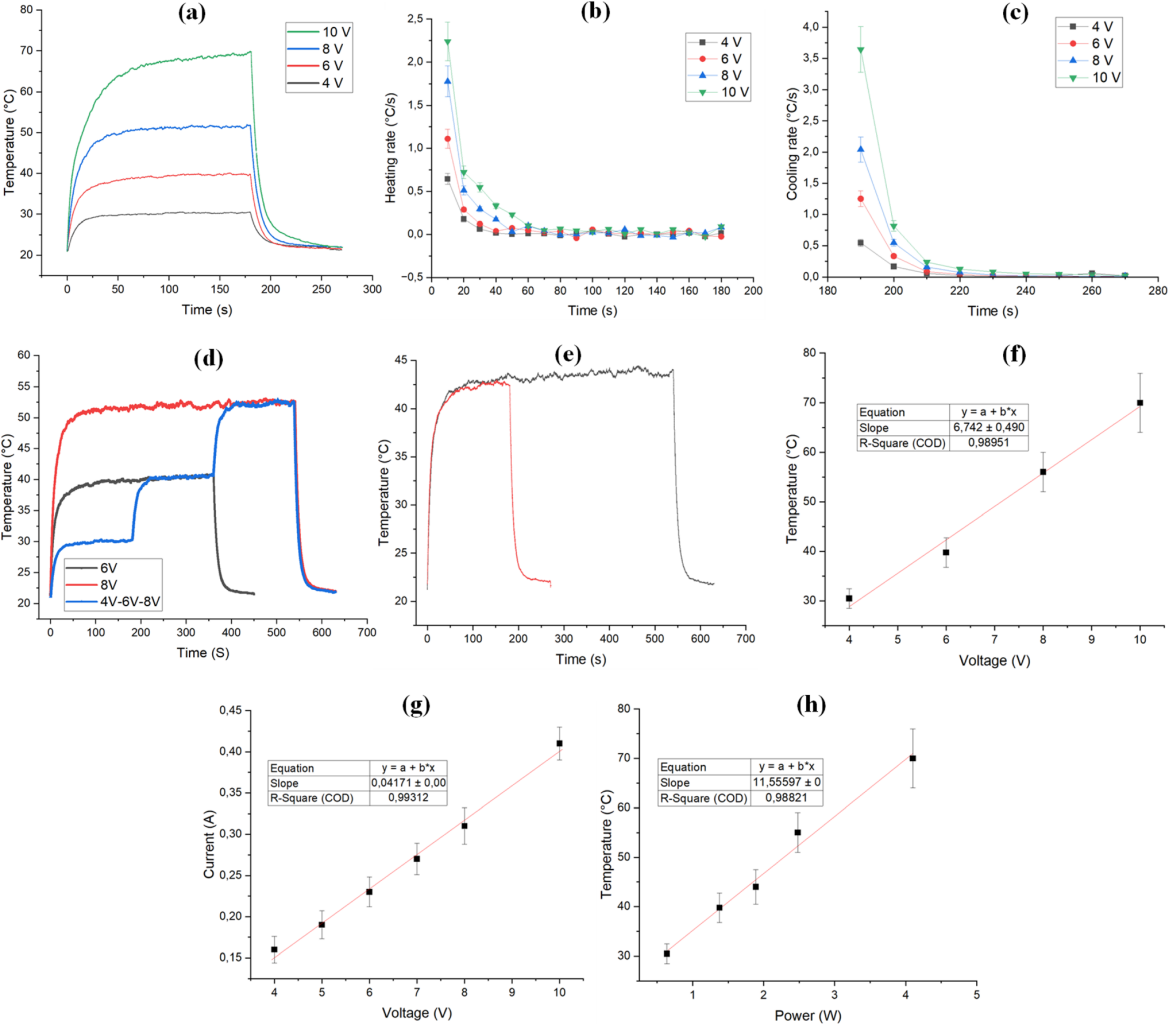
(a) Temperature *vs.* time curve under the applied voltages of 4 V, 6 V, 8 V, and 10 V, (b) heating rate after different applied voltages, (c) cooling rate after power source removal at different voltages, (d) accurate step-by-step temperature regulation with applied voltages, (e) fast response time and stable temperature profile during a long-time measurement, (f) maximum surface temperature *vs.* voltage curve under the relative humidity condition of 70%, (g) current *vs.* voltage curve for the coated sample, (h) maximum surface temperature *vs.* power curve under the relative humidity of 70%.

The electrical heating properties of the samples, analyzed using IR thermal imaging, are summarized in Fig. 2. The analysis was conducted under applied voltages ranging from 4 V to 10 V, with increments of 2 V. Fig. 2a illustrates the relationship between applied voltage and surface temperature. The heating process can be divided into three stages of heating stage, the steady-state stage, and the cooling stage. The surface temperature rose rapidly when the voltage was applied and dropped immediately after power was turned off at 180 seconds, demonstrating the rapid thermal response of the coated fabric. In addition, the surface temperature increased linearly with the increase in applied voltage, consistent with Joule's law.^50^ Using the first-order response time interpolation method reported by Zhou *et al.*,^51^ the estimated response time in this study ranged from 5 to 15 seconds. This is lower than the 30–40 s response time reported for single-walled CNT-functionalized fabric at applied voltages of 10 to 40 V.^52^ Similarly, coated textiles with polypyrrole exhibited response times exceeding 60 s in voltage ranges of 5 to 30 V, while those coated with PEDOT/PTSA showed similar response times in the 6 to 24 V range.^53,54^Fig. 2b shows the heating rate of the coated sample in every 10-second step cycle after the applied voltages of 4–10 V. The initial heating rate has a direct relation with the applied voltages where the higher applied voltages resulted in a higher initial heating rate. However, heating rate curves for all applied voltages showed approximately zero rate after 70 seconds, exhibiting the short period between the heating initiation and steady-state condition. Nanomaterial-based heaters typically exhibit longer heating response times due to substrate interference, which slows the measured heating rate compared to a free-standing setup.^51^ Notably, Liu *et al.* produced a hierarchically helical CNT fiber that possessed an ultrafast thermal response up to 1030 °C s^−1^ for the length of 2 cm under the applied voltage of 8 V. However, the temperature–time curves of heating textile woven from these CNT fibers and cotton threads (15 × 7 cm^2^) showed a maximum temperature of 60 °C under the applied voltage of 9 V after 100 s.^55^ Conversely, the incorporation of highly conductive fillers into textile fabrics may not result in very high electrical heating performance. This can be due to the fact that the binder affects the dispersion and adhesion of the conductive filler. Some binders may insulate or partially encapsulate the filler particles, limiting the available conductive pathways and reducing heating performance. If the conductive fillers are not well dispersed and tend to aggregate, the conductive pathways become less effective. Large agglomerates create localized conductivity variations, leading to inefficient heat distribution. The distribution and connectivity of fillers within the polymer matrix play a crucial role. If the percolation threshold is not sufficiently exceeded, or if the filler particles are not well connected, the overall electrical conductivity remains limited, reducing heating efficiency. Fig. 2c presents the cooling rate for the heated sample after the removal of the power source. The cooling rate for the heated sample under the higher applied voltages follows a rapid cooling rate. This could be due to the higher temperature difference between room temperature and the surface of the sample under the higher applied voltages. The higher temperature difference results in higher heat dissipation from the surface of the sample after the removal of the power source. In addition, it can be observed that the cooling rate reached the approximate cooling rate of zero after only 30 seconds of the removal of the power source. Fig. 2d highlights the successful regulation of temperature, with stepwise increases mirroring the original voltage increments. In addition, it shows the fast temperature response for the change in voltage in every step, allowing fast and accurate control of the temperature. Fig. 2e shows the coated pattern's stable temperature profile at 7 V over 9 minutes, reaching a steady state within 70 seconds. Fig. 2f–h show the relationships between, temperature, voltage, current, and power graphs for the coated pattern. There were strong linear correlations (*R*^2^ ≈ 0.99) between voltage and temperature, voltage and current, and temperature and power. This demonstrates the coated sample's efficient and predictable heating behavior.

### 3.3 Effect of relative humidity on joule heating

The relationship between clothing comfort and relative humidity (RH) is crucial, as RH impacts the moisture retention and heat dissipation properties of fabrics, which directly influence both thermal and perceived comfort. For electrically heated textiles, RH can affect thermal performance by altering the fabric's electrical conductivity. Excess moisture may reduce heating efficiency or lead to uneven heat distribution, ultimately compromising comfort.^42^

**Fig. 3:**
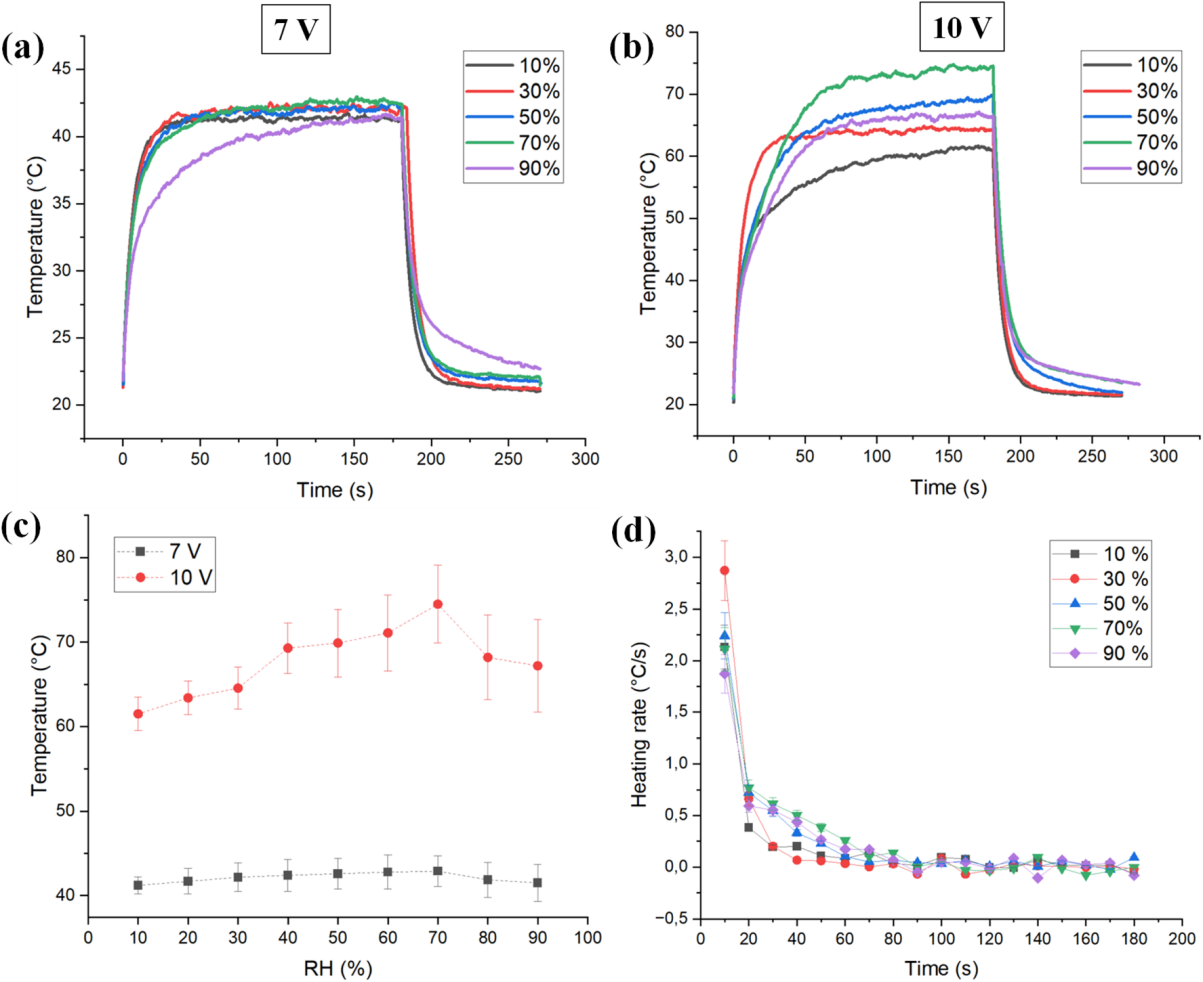
The effect of relative humidity conditions on the temperature *vs.* time curve under the applied voltages of (a) 7 V, and (b) 10 V in the room temperature of 20 °C (c) the relation between applied voltage and maximum surface temperature of the coated sample under different relative humidity conditions (d) the effect of relative humidity condition on the heating rate of the coated sample during the joule heating under the applied voltage of 10 V.

To assess the impact of RH on the electrical heating properties of the coated fabric, surface temperature changes were monitored under applied voltages of 7 V and 10 V at various RH% (Fig. 3a and b). The data reveal that RH significantly influences the electrical heating performance of the coated sample, with a more pronounced effect observed at the higher voltage of 10 V compared to 7 V. It seems that higher surface temperature will affect the moisture at close contact with the surface of the sample. This is more pronounced at higher temperatures. In addition, as voltage increases, joule heating intensifies, which may amplify the impact of humidity-induced changes. This could lead to a nonlinear response, where the heating efficiency is significantly influenced by RH at elevated voltage levels. Similar results have been reported where a more pronounced effect of RH on electrical heating properties was observed in higher applied voltages.^42^

Fig. 3c compares the maximum surface temperatures achieved under 7 V and 10 V across various RH levels. At both voltage levels, the lowest surface temperatures occurred at an RH of 10%. As RH increased, the surface temperature rose, peaking at 70% RH, before declining beyond this point. This could be due to the decrease of heat transfer with increasing relative humidity due to lower air thermal conductivity.^56^ In other words, the amount of heat dissipation from the surface of the heated sample has a direct relation with the thermal conductivity of the surrounding environment. Therefore, lower thermal conductivity will result in lower heat dissipation from the surface of the heated sample, which will finally result in a higher surface temperature for the heated sample. Notably, at 10 V, a temperature difference of 13 °C was observed between RH levels of 10% and 70%, whereas a smaller difference of 2.5 °C was recorded at 7 V. Meanwhile, in very high relative humidity (such as 90%), the relative humidity (RH) can affect electrical resistance due to moisture absorption by both the textile substrate and the hydrophilic components of the binder. In this case, water molecules can disrupt conductive pathways by swelling the coating matrix, which will result in increasing the inter-particle distance between carbon nanotubes and altering contact resistance. In addition, the absorbed moisture may form a dielectric layer around conductive fillers and deteriorate the efficiency of electron tunneling.^42^ The temperature–time profile of the coated sample under varying relative humidity (RH) conditions demonstrates that RH significantly impacts the heating rate. As shown in Fig. 3d, the heating rate was analyzed at 10-second intervals. The highest heating rate during the initial 10 seconds was observed at an RH of 30%, followed by a sharp decline in subsequent intervals. However, as RH increased from 30% to 90%, the initial heating rate during the first 10 seconds decreased. This could be simply due to the higher heat capacity of humid air compared to dry air, which results in higher energy to heat the humid air. Notably, while an RH of 70% enhanced the maximum temperature achieved, it also led to a reduced initial heating rate. These findings indicate that while higher RH levels can improve peak heating performance, they may simultaneously dampen the initial heating response. These findings highlight the significant influence of RH on the heating performance of electrical textiles.

### 3.4 Effect of ambient temperature (AT) on joule heating

**Fig. 4:**
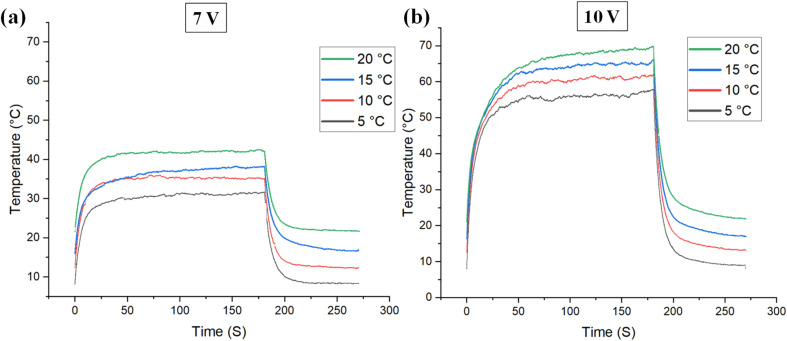
Effect of ambient temperature on the surface temperature *vs.* time profile of the sample under the applied voltages of (a) 7 V, and (b) 10 V and relative humidity of 50%.

Fig. 4 illustrates the impact of AT on the Joule heating properties of the coated patterns under a constant relative humidity of 50% and applied voltages of 7 and 10 V. A reduction in AT caused a decrease in the surface temperature of the coating at both voltage levels. This behavior can be attributed to the increased temperature gradient between the heating textile's surface and the surrounding air as the environmental temperature drops. The rate of heat transfer is directly proportional to the temperature gradient; a larger gradient leads to faster heat loss, whereas a smaller gradient slows down heat transfer.^57,58^ For instance, under an applied voltage of 7 V, the surface temperature decreased from 43 °C to 30 °C as the environmental temperature dropped from 20 °C to 5 °C (Fig. 4b). In addition, AT also plays an important role by affecting polymer segmental mobility, which can influence the rearrangement of conductive pathways. At elevated temperatures, enhanced molecular motion may improve contact between conductive particles, thus reducing resistance. However, excessive thermal expansion or softening of the binder may also degrade interfacial adhesion over time. A further decrease in environmental temperature would likely result in even lower surface temperatures, potentially limiting the effectiveness of heating textiles in extreme conditions. These findings emphasize the critical role of AT as a key factor in the performance of heating textiles. In addition, it highlights the importance of insulation for minimizing heat loss. Proper insulation should still be the first layer of protection against extreme cold, while electrically heated textiles can offer additional warmth.

### 3.5 Impact of insulation layers on electrical heating performance

The primary requirement for an effective heating textile is to provide uniform heating with minimal heat loss. The heat generated within a fabric or structure can transfer internally or through the body and dissipate into the surrounding environment.^59^ To ensure efficient heating, the key principle is to minimize the loss of body heat or generated heat to the environment.^60^ Achieving this requires the use of materials with excellent thermal insulation properties.^61^ Such materials should retain heat or reflect it back to the body by infrared heat radiation or minimize the heat loss.^60^

**Fig. 5:**
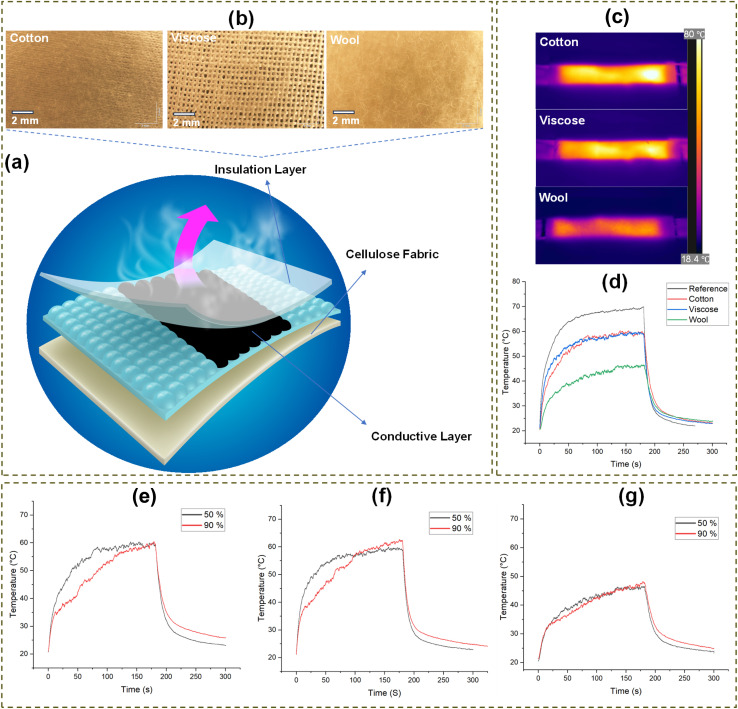
(a) Schematic design of the heating pad with joule heating method and insulation layer, (b) the optical microscopy images of the surface of the cotton, viscose, and wool as the layering fabrics (c) the thermal IR image of the surface of the layering fabrics under the relative humidity of 50% and applied voltage of 10 V (d) surface temperature profile of the layering fabrics (cotton, viscose, and wool) on top of the heating coated sample under the relative humidity conditions of 50% and applied voltage of 10 V in comparison with reference heated sample. The temperature *vs.* time profile of the (e) cotton, (f) viscose, (g) wool under two relative humidity conditions of 50% and 90% and applied voltage of 10 V.

Three different fabric types (*i.e.*, cotton, viscose, and wool) were placed on the coated fabric to investigate their insulation properties and assess their effectiveness in retaining heat for efficient heating applications (Fig. S2). Fig. 5a presents the schematic design of the heating pad with the Joule heating method and insulation layer for effective heat maintenance. Fig. 5b shows the optical microscopy images of the cotton, viscose, and wool fabrics as the insulation layers covering the surface of the Joule heating-coated pattern. Fig. 5c shows the IR images of the coated pattern after layering with different fabrics under the applied voltages of 10 V and relative humidity of 50%. The images illustrate heat transfer through cotton, viscose, and wool, with wool demonstrating superior insulation by effectively retaining heat, making it the most efficient for electrical heating applications. Fig. 5d shows the surface temperature profile of these layering fabrics on top of the electrical heating pattern under the applied voltage of 10 V and relative humidity of 50%. Furthermore, the study compares the heating profile of the fabric surface with different insulating layers to that of the heated fabric without insulation, used as a reference sample. The results indicate that the heating profile varies depending on the type of insulation fabric. Specifically, the maximum surface temperature of cotton- and viscose-insulated fabrics was 11 °C lower than that of the reference coated fabric, while the wool-insulated fabric exhibited an even greater reduction of 24 °C. Additionally, the wool-insulated fabric demonstrated a slower heating rate, further emphasizing its superior insulating properties.

Fig. 5f–h show the temperature–time curve of the layering fabric in two different relative humidity conditions of 50% and 90%. It can be seen that the maximum surface temperature after 3 minutes for all three types of fabrics increases. This effect can be attributed to water molecules replacing air in high-humidity conditions. Since water has a significantly higher thermal conductivity (0.6 W m^−1^ K^−1^) compared to air (0.026 W m^−1^ K^−1^ at 20 °C), this substitution enhances heat transfer within the fabric structure. The highest increase was seen in viscose since it had a higher pore area (Table 2) and, therefore, a higher content of air was replaced with water, affecting the thermal conductivity to a higher extent. Similar trends of increased thermal conductivity with moisture content have been reported in other research.^62^ The moisture regain of all three types of fabric in both relative humidity conditions of 50% and 90% has been reported in Table 2. It can be seen that the viscose fabric with a high pore area presented moisture regain values higher than cotton and similar to wool. In addition, in the relative humidity condition of 90%, the moisture regain increased nearly twice the value compared to the relative humidity condition of 50%. While the temperature *vs.* time curves for the insulators in different relative humidity conditions revealed a higher maximum surface temperature achieved under the relative humidity condition of 90% compared to 50%, the heating rate under higher relative humidity values is lower. This may be attributed to the fabrics absorbing higher water content under increased relative humidity, which adds to the weight of the insulators, similar to having a thin layer of water on the heating surface. Part of the heat is used to evaporate the water, delaying the heating of the insulator and affecting the overall heating rate.

**Table 2:** The thickness, pore area percentage, and moisture regain of the cotton, viscose, and wool as the layering fabrics

Fabric	Pore area (%)	Moisture regain (%) after 1 h at 50% RH	Moisture regain (%) after 1 h at 90% RH
Cotton	2.4 ± 0.7	8.5	15.9
Viscose	32.1 ± 2.3	10.6	21
Wool	0.9 ± 0.5	11.5	23.7

Textile thermal properties (*i.e.*, resistance, conductivity, and absorptivity) are generally influenced by a series of factors including fabric structure, density, humidity, fiber type, weave, surface treatments, compressibility, air permeability, and ambient conditions.^62^

**Fig. 6:**
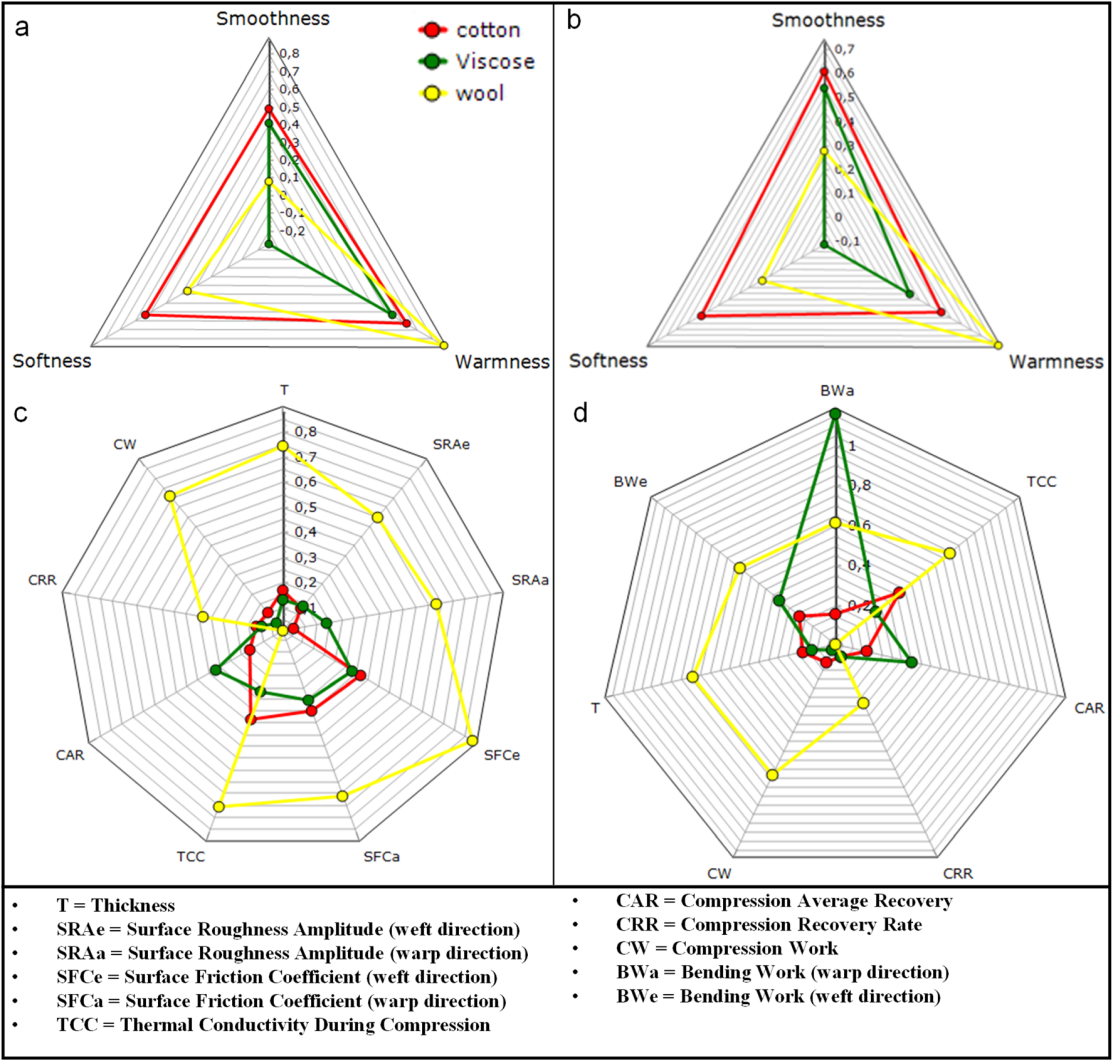
Primary sensorial indexes for (a) face and (b) back sides and (c and d) their corresponding thermal indices of layered fabrics.

The impact of thermal transport and mechanics of the insulator specimens was further quantified by the FTT test, illustrated in Table 3. The peak heat flux (*Q*_max_) shows the instant heat transfer at contact. Cotton has the highest *Q*_max_ with a value of 1064 and 1105 W m^−2^ in the face and back side of the sample, respectively. The higher *Q*_max_ produces a stronger initial thermal sensation and a steeper early IR slope. The IR slope in viscose is slightly lower, which corresponds to a lower *Q*_max_ value compared to cotton. Wool has the lowest *Q*_max_ with a value of 640 W m^−2^ in its outer and 685 W m^−2^ inner surfaces. This explains the rationale behind warm/cool contact^63^ and explains the primary reason wool feels less hot at first even when the heater runs warmer internally.^63–66^ As a result, it has the highest warmness value of 0.73 compared to 0.46 for cotton and 0.32 for viscose as shown in Fig. 6.

**Table 3:** Summary of FTT indices including bending, compression, heat flux, friction, and surface roughness^a^

Sample type	Side	Bending		Compression			Heat flux			Friction		Roughness	
		BWa	BWe	*T* (mm)	CW (gf mm)	CRR	TCC	TCR	*Q*_max_ (W m^−2^)	SFCa	SFCe	SRAa	SRAe
		(gf mm rad)					W mm m^−2^ C^−1^					µm	
Cotton	Face	446.39	535.09	0.36	350.34	0.26	37.57	37.19	1063.72	0.30	0.31	15.36	32.74
	Back	391.09	578.79	0.36	362.43	0.22	39.93	38.72	1104.80	0.34	0.28	14.49	26.98
Viscose	Face	3326.87	927.09	0.29	209.94	0.24	29.86	29.35	1012.95	0.28	0.29	47.32	35.96
	Back	2826.56	889.18	0.29	193.47	0.22	29.88	29.38	1034.11	0.30	0.25	37.03	43.94
Wool	Face	1506.15	1469.93	1.30	1898.91	0.44	61.98	60.86	639.14	0.51	0.61	155.05	147.01
	Back	1500.48	1514.67	1.30	1947.13	0.44	61.10	60.52	684.51	0.63	0.53	154.12	130.09

a 1 gf mm rad = 9.806 × 10^−6^ N m rad.

The interfacial conductance is affected by the real area of contact^63,67^ at both interfaces, which is impacted by surface roughness and friction. Wool shows the highest roughness amplitude (SRA) 130–155 µm and friction (SFC) 0.5–0.63 followed by viscose. A napped wool face touches mainly at tuft tops of fiber tips, and most of the nominal area is separated by interfacial micro-gaps. Hence, heat has to pass through sparse micro-contacts and across air gaps, which will affect heat and vapor transfer from the guarded hot plate surface. It has also been studied in previous research^64^ that the time delay caused by the propagation of the heat wave front within the specimen becomes very small when the thickness of the specimen is small. Wool exhibited a thickness of 1.30 mm, which is more than four times greater than viscose, which has a thickness of 0.29 mm, as shown in Table 3. The resulting constriction resistance lowers the first-second flux and slows the outer-surface rise in IR. In contrast, the smoother surface in cotton closes micro-gaps and increases real contact. As a result, outward heat flow starts higher, and the outer surface lets heat flow out faster. These mechanisms follow classic contact-resistance models for rough surfaces illustrated in previous literature.^68–71^

Steady heat loss depends on how much thickness the cover layer maintains under small loads. This behavior can be related to compression work (CW) and compression recovery rate (CRR). Wool shows CW 1899–1947 gf mm and CRR 0.44 at about 1.30 mm thickness (*T*). High CW and recovery in wool preserve a thicker still air layer under applied loads of 0–8470 gf. When the cover keeps its thickness, less heat leaks through it. More of the input stays near the heater, so the inner side runs hotter while the outside surface is cooler at the same electrical power. Cotton and viscose have CW 350–362 and 193–210 gf mm with CRR approximately 0.22–0.26 at 0.36 mm and 0.29 mm, respectively. It shows that they flatten more, and the air gap shrinks, and the steady loss increases as a result. This aligns with clothing studies^72^ showing insulation rises with stable air gaps and falls when gaps collapse or are ventilated. In practice, a high CW cover can reach the same comfort level with less electrical input.

Bending work describes how closely the cover layer follows the heater under light pressure. Low bending work in thin knit cotton promotes conformity and closes interfacial micro-voids. This increases the real contact area, which increases interface conductance, which could speed up the warming of the outer surface. High bending work in napped wool tends to bridge small undulations and leaves micro-voids. This could allow for dropping conductance to warm the outer surface warms more slowly. Drape and fit studies^73^ show that bending rigidity and garment fit change the contact area and air-gap distribution, which enhances the heat transfer. The thermal channel measured under load shows the same pattern. The thermal conductivity recorded during compression (TCC) was about 61 W mm m^−2^ C^−1^ for wool, 37–40 W mm m^−2^ C^−1^ for cotton, and 29–30 W mm m^−2^ C^−1^ for viscose. Thermal conductivity during recovery (TCR) also followed the same pattern. In this stacked heater system, those values reflect the resistive route set by preserved loft under a defined pressure, which is not a contradiction of the cooler outer surface seen for wool at equal power.

Overall, the smooth and pliable cotton cover makes broad contact and channels heat out quickly. It feels warm immediately but loses more heat at the same power. Viscose follows a nearly similar pattern to cotton, shown in Fig. 6. The lofty, napped wool cover keeps a thicker air layer and imperfect contact. As a result, the pad holds heat inside and the outer face stays cooler, which matches the IR curves.^65,68,72^ In practice, cotton can be chosen as the contact layer to skin, and wool can be selected as an energy-efficient heat retention insulator as the outer layer in contact with the environment.

### 3.6 Sensitivity to temperature

**Fig. 7:**
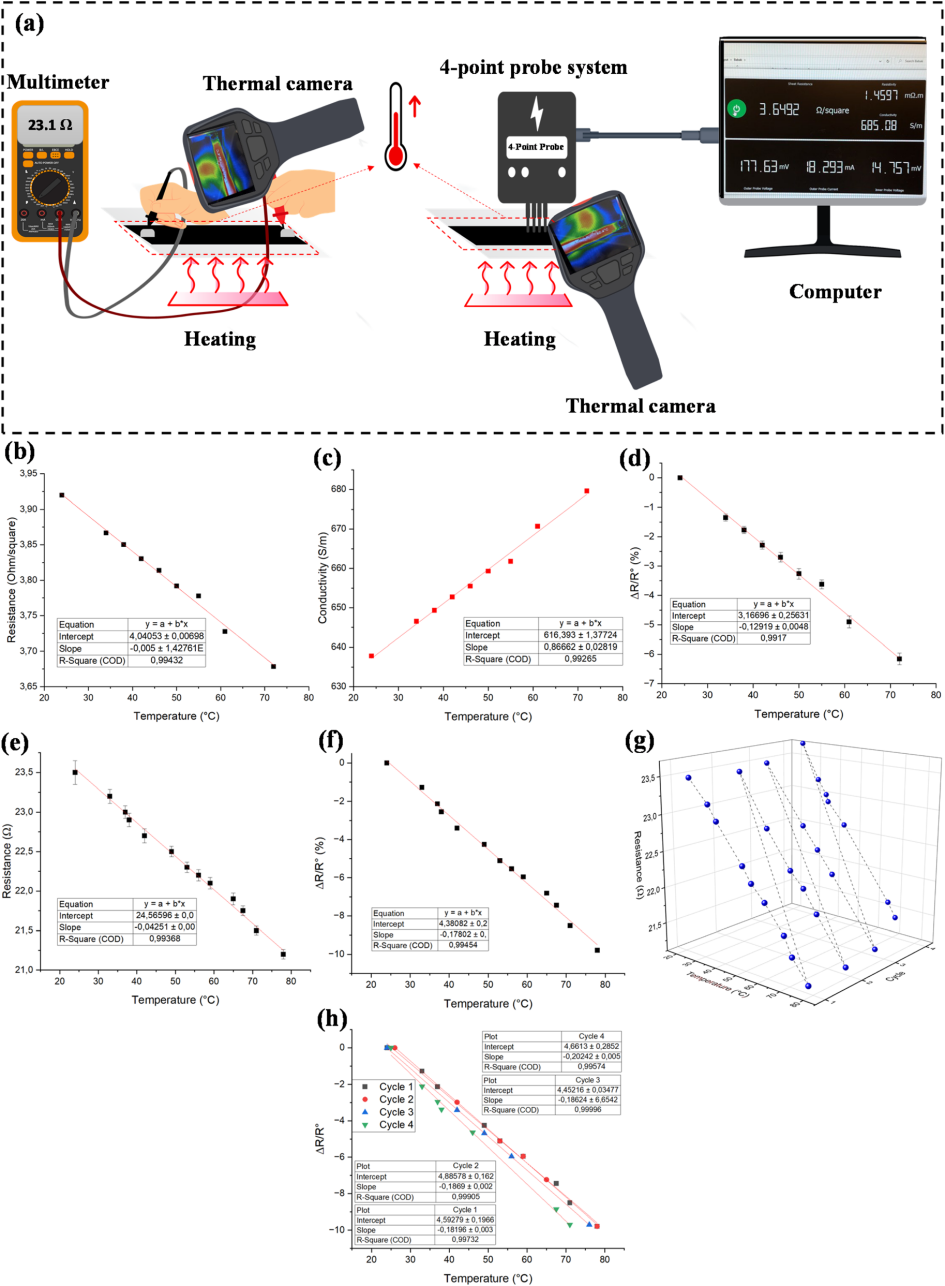
(a) Schematic design of temperature sensing process using two different approaches, (b) sheet resistance, (c) conductivity change, and (d) resistance change per initial resistance *versus* rising temperature for the individual spot-by-spot responsivity measurement. (e) Resistance and (f) resistance change per initial resistance *versus* temperature for the whole surface responsivity measurement, (g) cyclic temperature sensing performance of the whole surface, (h) repeatability of the temperature sensing during four heating and cooling cycles.

Integrating temperature sensors with heating fabrics creates a responsive system where real-time temperature monitoring guides heating adjustments, forming a feedback loop that enhances comfort and energy efficiency. To evaluate this, the electrical responsivity of the sample was examined using two approaches. First, the electrical responsivity of the individual spots has been analyzed. In this approach, the electrical properties of the individual spots of the sample were monitored during different temperature setups. Secondly, the electrical responsivity of the whole sample has been studied. Fig. 7a shows the schematic design of the experiment for temperature sensing through two different approaches. Accordingly, the resistivity of the whole sample was monitored at various temperatures. Fig. 7b and c show the change in sheet resistance and conductivity with temperature change for the spot responsivity of the sample. It can be seen that in this temperature region, the resistance and conductivity changed almost linearly with the change in temperature, with the linearities of 0.9943 and 0.9926. In addition, the changes in resistance were observed immediately for the sample in contact with heat, highlighting the fast response time for the designed temperature sensor. Fig. 7d shows the resistance change per initial resistance *versus* rising temperature for the spot responsivity measurement. The change in electrical resistance is directly related to the Temperature Coefficient of Resistance (TCR). TCR is defined as the relative change in resistance per unit temperature change (1 K), where *R*_0_ represents the initial resistance. TCR is a critical parameter for assessing the thermal sensitivity of temperature sensors. Notably, the TCR of materials increases with higher hard segment content and greater molecular weight of soft segments. Here, the TCR value of the pattern with the spot control was 0.13% K^−1^. Fig. 7e represents the change in resistance with an increase in temperature for the whole length of the pattern. Similarly, the resistance followed an almost linear behavior toward changes in temperature (*r*^2^ = 0.9945). Fig. 7f displays the change in resistance percentage per initial resistance with the temperature increase. The TCR value for this approach was 0.18% K^−1^. It can be concluded that the responsivity using the whole sample was better than spot-by-spot responsivity. In addition, the sensing performance of the pattern was investigated in 4 cycles of cooling and heating. Fig. 7g shows the cyclic sensing performance of the whole pattern. It can be seen that the sample demonstrated excellent repeatability following the trend of cooling and heating. Fig. 7h demonstrates the slope and the linearity of the sensing sample after 4 cycles of heating and cooling. The mean slope value for the 4 cycles was 0.189 ± 0.007 with *R*^2^ = 0.9980, demonstrating the excellent repeatability of the designed temperature sensor. Although only four heating–cooling cycles were performed in this study, the test was intended as a preliminary evaluation of sensing stability. Our results exhibited negative TCR values, aligning with previous studies that utilized MWCNTs as temperature-sensitive conductors.^74–78^ This could be due to the fact that in MWCNTs, electrical conductance occurs through delocalized π electrons, which transfer between neighboring nanotubes *via* the tunneling effect. As the temperature increases, thermal excitation promotes electron hopping and tunneling between nanotubes, reducing junction resistance and improving conductivity. The reduction of contact resistance at tube junctions and potential thermal-induced reorientation of nanotubes, improving percolation pathways, can also contribute to this behavior. These factors collectively lead to a decrease in resistance and an overall increase in conductivity with temperature.^79^ However, it is important to note that some studies have also reported positive TCR behavior in MWCNT-based temperature sensors.^80^ This discrepancy suggests that the conduction mechanism is highly dependent on material composition and processing conditions. A positive TCR can result from polymer matrix expansion, which increases inter-nanotube distances and disrupts conduction, low CNT loading, leading to an unstable network, and structural defects or oxidation, which introduce charge transport barriers and enhance thermal sensitivity. These observations highlight the complex interplay between tunneling conduction, nanotube junction behavior, and matrix effects in MWCNT-based systems. The TCR value reported in this work is in a similar range to those previously reported values.^74,76,77,80^ However, polymeric-based films as substrates offer slightly higher sensitivity in comparison with textile fabric. To explain the large TCR observed, three main factors have been identified based on the previously described conductive mechanism: percolation theory, the stretchability of conductive particles, and the thermal expansion characteristics of the polymer matrix.^81,82^ First, the percolation effect was exploited to enhance sensitivity by selecting filler volume fractions close to the percolation threshold, where small structural changes cause significant resistance variations.^81,83,84^ Second, the type and morphology of conductive fillers influence the TCR. For example, carbon nanofibers (CNFs), owing to their high aspect ratio, dispersed in insulating PDMS, can yield higher sensitivity because thermal expansion separates multiple conductive pathways, increasing resistance. Compared with CB and CNFs, graphite powder has been reported to produce even greater temperature sensitivity.^85^ Third, using semicrystalline polymers can also boost sensitivity, as these materials undergo a crystalline-to-amorphous phase transition near their melting point, leading to significant volume expansion. This increases interparticle distances, thereby altering conductivity.^86^ Together, these effects enhance the sensitivity of polymer-based sensors, allowing for simplified readout circuitry without the need for per-pixel amplification before data acquisition.^87^

Polymeric sensors incorporating semicrystalline polymers are typically limited to a narrow operational temperature range, which restricts their suitability for wide-scale temperature sensing. Moreover, single-polymer systems often exhibit poor reproducibility of resistivity, temperature relationships, with stability frequently limited to fewer than 100 thermal cycles.^81,88^ This instability is primarily attributed to morphological changes in the conductive network, as most conductive fillers lack chemical bonding to the polymer matrix or to each other, preventing the resistivity from fully returning to its original value after cooling.^81,86^ In addition, improving measurement accuracy, response time, and minimizing hysteresis remain significant challenges for polymeric sensors.^81,89^ A potential route for future improvement involves tailoring the morphology of the polymer matrix and optimizing polymer domain sizes.^86^

## 4. Conclusions

This study investigated the electrical heating performance, environmental adaptability, and temperature sensing capabilities of a cellulose fabric coated with a formulation containing MWCNT and two different binders. The results revealed that the surface temperature of the samples increased proportionally with applied voltage, achieving rapid heating and stable temperature profiles, in line with Joule's law. Environmental factors such as relative humidity (RH) and ambient temperature were shown to influence the heating performance. The optimal RH for achieving maximum surface temperature was 70%. Conversely, decreasing ambient temperature led to faster heat dissipation, underscoring the role of environmental conditions in heating textile performance. Layering fabrics with different insulation properties highlighted the critical role of thermal insulation in heat retention. In practice, the smooth and pliable cotton fabric can be chosen as the contact layer to skin, and wool can be selected as an energy-efficient heat retention insulator as the outer layer in contact with the environment, while viscose, which shows lower warmth compared to wool and cotton and lower smoothness and softness compared to cotton, may be less suitable to be either the contact layer or the insulator. Furthermore, the temperature sensing capabilities of the samples showed excellent responsiveness and repeatability, with consistent cyclic performance in the range of 20 to 80 °C. The integration of heating and sensing functions highlights the potential for smart textile applications in adaptive heating systems. Overall, this work provides a comprehensive understanding of the interplay between material composition, environmental factors, and functional performance, paving the way for the development of advanced smart textiles for wearable medical and therapeutic devices, cold-weather protective clothing, and smart heating systems.

## Author contributions

Babak Abdi: methodology, software, validation, formal analysis, investigation, data curation, writing – original draft, writing – review & editing, visualization; Esubalew Kasaw Gebeyehu: methodology, formal analysis, investigation, data curation, writing – review & editing; and Ali Tehrani-Bagha: conceptualization, methodology, formal analysis, resources, writing – review & editing, supervision, project administration and funding acquisition.

## Conflicts of interest

There are no conflicts of interest to declare.

## Supplementary Material

RA-015-D5RA07103H-s001

## Data Availability

The data that support the findings of this study are available from the authors on reasonable request. Supplementary information (SI) is available. See DOI: https://doi.org/10.1039/d5ra07103h.
