# Supplementary material for: MWCNT-coated textiles for heating and temperature sensing under varying environmental conditions
Source: RSC Adv. 2025 Dec 8;15(56):47735–50. doi: 10.1039/d5ra07103h (PMC12683328; doi:10.1039/d5ra07103h)
Supplement: RA-015-D5RA07103H-s001 [file RA-015-D5RA07103H-s001.pdf]

Supplementary Information (SI) for RSC Advances.
This journal is © The Royal Society of Chemistry 2025

Supporting Information for

MWCNT-Coated Textiles for Heating and Temperature Sensing under Varying Environmental Conditions

Babak Abdi, Esubalew Kasaw Gebeyehu, Ali R. Tehrani-Bagha*
School of Chemical Engineering, Aalto University, Espoo 02150, Finland
* Corresponding author: ali.tehrani@aalto.fi

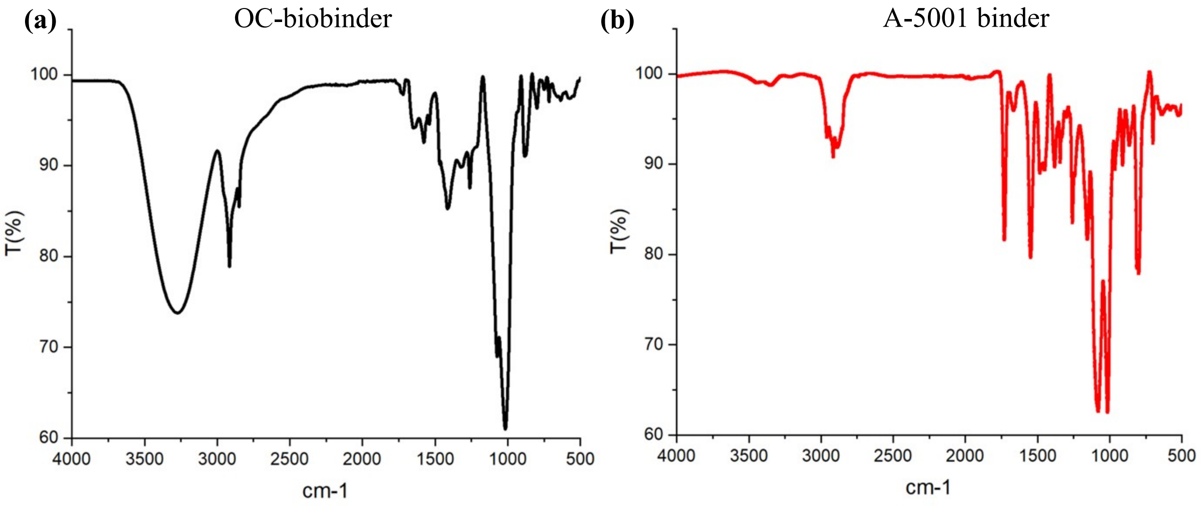

Figure S1. ATR-FTIR curve of the (a) OC-biobinder, and (b) ATR-FTIR curve of A-5001 binder.

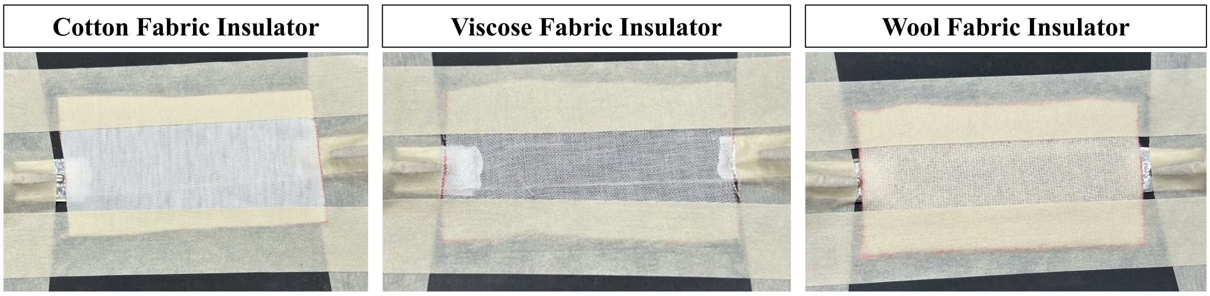

Figure S2. The attachment of the fabric insulators to the Joule heating pattern.
